# Rodent Models of Alcoholic Liver Disease: Role of Binge Ethanol Administration

**DOI:** 10.3390/biom8010003

**Published:** 2018-01-13

**Authors:** Shubha Ghosh Dastidar, Jeffrey B. Warner, Dennis R. Warner, Craig J. McClain, Irina A. Kirpich

**Affiliations:** 1 Division of Gastroenterology, Hepatology and Nutrition, Department of Medicine, University of Louisville School of Medicine, Louisville, KY 40202, USA; s0ghos05@louisville.edu (S.G.D.); jbwarn01@louisville.edu (J.B.W.); dennis.warner@louisville.edu (D.R.W.); craig.mcclain@louisville.edu (C.J.M.); 2 Department of Pharmacology and Toxicology, University of Louisville School of Medicine, Louisville, KY 40202, USA; 3 Robley Rex Veterans Medical Center, Louisville, KY 40202, USA; 4 University of Louisville Alcohol Research Center and Hepatobiology & Toxicology COBRE, University of Louisville, Louisville, KY 40202, USA

**Keywords:** alcoholic liver disease, animal models, EtOH-induced liver injury, binge drinking, blood alcohol concentration, high fat diet, lipopolysaccharide

## Abstract

Both chronic and acute (binge) alcohol drinking are important health and economic concerns worldwide and prominent risk factors for the development of alcoholic liver disease (ALD). There are no FDA-approved medications to prevent or to treat any stage of ALD. Therefore, discovery of novel therapeutic strategies remains a critical need for patients with ALD. Relevant experimental animal models that simulate human drinking patterns and mimic the spectrum and severity of alcohol-induced liver pathology in humans are critical to our ability to identify new mechanisms and therapeutic targets. There are several animal models currently in use, including the most widely utilized chronic ad libitum ethanol (EtOH) feeding (Lieber–DeCarli liquid diet model), chronic intragastric EtOH administration (Tsukamoto–French model), and chronic-plus-binge EtOH challenge (Bin Gao—National Institute on Alcohol Abuse and Alcoholism (NIAAA) model). This review provides an overview of recent advances in rodent models of binge EtOH administration which help to recapitulate different features and etiologies of progressive ALD. These models include EtOH binge alone, and EtOH binge coupled with chronic EtOH intake, a high fat diet, or endotoxin challenge. We analyze the strengths, limitations, and translational relevance of these models, as well as summarize the liver injury outcomes and mechanistic insights. We further discuss the application(s) of binge EtOH models in examining alcohol-induced multi-organ pathology, sex- and age-related differences, as well as circadian rhythm disruption.

## 1. Introduction

Alcohol consumption, both acute and chronic, is an important social, economic, and clinical problem. The harmful use of alcohol ranks as the fifth leading global cause of preventable morbidity and mortality [1,2,3]. According to the National Epidemiologic Survey on Alcohol and Related Conditions III (NESARC), the 12-month and lifetime prevalence of alcohol use disorders were 13.9% and 29.1%, respectively, among US adults aged 18 and older [4]. The prevalence of these two parameters was generally higher for males (17.6% and 36.0%, respectively) compared to females (10.4% and 22.7%, respectively). 

Excessive alcohol intake is a causal factor in a wide range of multi-organ pathology, including alcoholic liver disease (ALD). ALD is manifested as a spectrum of clinical disorders ranging from steatosis (fatty liver) to alcoholic hepatitis (AH, a combination of steatosis and inflammation), and may progress further to the more severe forms, cirrhosis and hepatocellular carcinoma. AH is associated with high mortality; up to 40% of severe AH patients die within six months [5]. However, there is currently no US Food and Drug Administration (FDA)-approved therapy for any stage of ALD. Therefore, the discovery of novel therapeutic strategies remains a critical need for patients with ALD. It is noteworthy that although most (~95%) heavy drinkers develop fatty liver, only a 20–40% subset of patients progresses to AH, and about 10–15% develop frank cirrhosis [4,5,6]. This suggests that additional factors such as sex, genetic, epigenetic, and environmental influences (e.g., diet, smoking, toxicant exposure) likely contribute to the pathogenesis of and inter-individual susceptibility to progressive ALD [7,8,9]. Further, one’s drinking pattern is a critical determinant of alcohol-induced liver damage [10,11,12,13,14,15,16]. In humans, alcohol intake can be acute (single occasion over the course of several hours), short-term (for several days), or long-term/chronic (for years/decades). The amount and duration of alcohol consumption correlates with the severity of liver disease [14,16]. Additionally, humans often indulge in long-term consumption intermixed with patterns of relative abstinence followed by 1–2 days of heavy alcohol consumption over the course of many years (i.e., chronic episodic binge). Many AH patients have a background of chronic drinking concomitant with a history of recent excessive alcohol intake [17,18,19]. Understanding how drinking patterns affect ALD initiation and progression might provide new insights into the molecular mechanisms, as well as help to design novel effective therapies. Experimental animal models that effectively reproduce human drinking patterns and mimic the spectrum and severity of alcohol-induced liver injury in humans are a necessity. 

Rodents (rats and mice) are the most commonly used animal models to study ALD. In general, experimental ALD is induced by ethanol (EtOH) administration in combination with dietary, chemical, or genetic manipulations. There are several animal models currently in use, including the most widely utilized chronic *ad libitum* EtOH feeding (Lieber–DeCarli liquid diet; LD diet), chronic intragastric (IG) EtOH administration model (Tsukamoto–French model), and second-hit models that combine EtOH administration with an additional hit(s) (e.g., a high fat diet (HFD), lipopolysaccharide, genetic knockout/overexpression, and others) to facilitate progression to advanced ALD. However, current animal models do not recapitulate the full spectrum of human ALD. Lieber–DeCarli *ad libitum* EtOH feeding (even for longer periods) usually causes hepatic steatosis with limited liver injury, inflammation and no fibrosis. The Tsukamoto–French IG EtOH feeding causes severe steatosis with inflammation and mild fibrosis, however, it is a technically complicated and labor-intensive model. The chronic-plus-binge EtOH administration paradigm recently developed at the National Institute on Alcohol Abuse and Alcoholism (NIAAA) of the National Institutes of Health (NIH) by Dr. Bin Gao simulates the drinking pattern(s) of heavy drinkers who indulge in chronic-binge-drinking and induces a robust neutrophil-mediated liver injury [20]. Given that hepatic neutrophil infiltration is a prominent clinical feature of AH [5,21,22] and correlates with disease prognosis [23], this paradigm demonstrates that incorporating a binge EtOH challenge in animal models can recapitulate certain components of the human disease and can help delineate mechanism(s) contributing to the development of progressive ALD.

**Figure 1:**
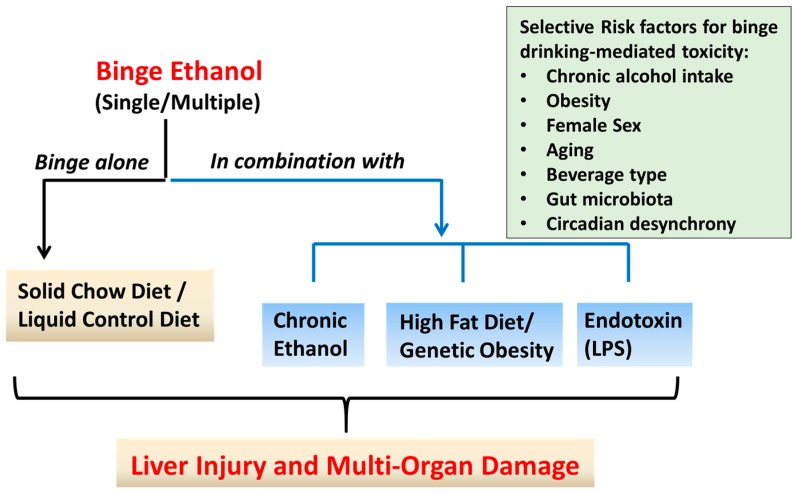
Paradigms of binge ethanol (EtOH) administration in rodent models. Approaches that have been used to model binge (acute) EtOH effects in rodents are shown. These include administration of single or multiple EtOH binge(s) alone, or in combination with short- or long-term exposure to chronic EtOH or high fat diet, or lipopolysaccharide (LPS) injection. Binge EtOH produces dose-, frequency-, and duration-dependent adverse effects on the liver and multiple organ systems. Factors that exacerbate binge alcohol-mediated toxicity in humans and rodent models are shown in the box.

Herein, we discuss the binge alcohol drinking pattern, the effects of binge EtOH intake on blood alcohol concentration (BAC), and review selected studies utilizing current rodent models of ALD that employ binge EtOH administration alone or in combination with chronic EtOH intake, HFD, or endotoxin challenge (Figure 1). Several routes of EtOH administration simulate binge drinking in rodent models, such as oral self-administration (e.g., two-bottle preference method), intraperitoneal or intravenous injection, oral gavage, intragastric infusion, etc. In this review, we have focused on studies where rodents were administered alcohol by oral gavage, which is the more commonly-used, physiologically relevant method of alcohol binge(s). We analyze the strengths, limitations, and translational relevance of these models, as well as summarize the liver injury outcomes and mechanistic insights (Table 1). We further discuss the application(s) of binge EtOH models in examining alcohol-induced multi-organ pathology, sex- and age-related differences, as well as circadian rhythm disruption.

## 2. Binge Alcohol Drinking Pattern and Blood Alcohol Concentration

### 2.1. Binge Drinking in Humans: Definition and Statistics

Binge drinking is defined by the NIH NIAAA as a pattern of alcohol drinking that brings BAC levels to 0.08% (80 mg/dL) or above, and which, for the typical adult, corresponds to consuming five or more drinks for males and four or more drinks for females in about 2h [38]. For assessing alcohol-related health risks in humans, the definition of a ‘standard drink’ is an important consideration. In the US, one ‘standard’ drink is considered to be one that contains ~14 grams of pure alcohol, which is found in 12 ounces of regular beer (~5% alcohol), 5 ounces of wine (~12% alcohol), 8–9 ounces of malt liquor (~7% alcohol), and 1.5 ounces of distilled spirits (~40% alcohol) [39]. 

Binge drinking is a significant economic and health concern and it accounts for approximately one-half of all alcohol-attributable deaths and incurs substantial economic costs [40,41,42]. Binge drinking was responsible for 77% of the total cost of alcohol misuse, or $191 billion, in 2010 [43]. The 2012–2013 NESARC indicates that among the 73% of adults who drank in the past year, 46% binged at least once [4]. Binge prevalence is especially high among adolescent and young adult populations with an estimated 37.9% of college students aged 18–22 years reporting binge drinking in the past month and 32.6% of other persons (non-college) of the same age [44]. 

### 2.2. Binge Alcohol Administration: Effects on Blood Alcohol Levels in Rodents

One of the major challenges in developing clinically relevant animal models of ALD is that rodents are inherently averse to self-administering EtOH in amounts required to produce significant increases in BAC and liver injury relevant to the human disease state. Given that the alcohol catabolism rate is up to five-times higher in rodents compared to humans, the amount of alcohol administered to animals to achieve sustained BAC and subsequent liver injury cannot be directly compared with human alcohol consumption [45]. In general, chronic *ad libitum* Lieber–DeCarli EtOH feeding in rodents (~4–6% EtOH *v*/*v* or *w*/*v*, with an estimated EtOH consumption of ~10–20 g/kg/day) produces BAC levels typically in the range of ≈80–160 mg/dL and induces mild liver injury [46,47,48,49,50,51,52]. The Tsukamoto–French IG alcohol infusion model was developed to overcome the natural aversion of rodents to EtOH consumption [53,54,55,56]. In rats, this model (8–12 g/kg/day of IG EtOH) achieves an average BAC of ~200 mg/dL concomitant with more severe liver injury including fibrosis [53,55]. Similarly, an IG EtOH infusion paradigm in mice, wherein EtOH doses were gradually increased over four weeks (22–35 g/kg/day), caused an average BAC of ~300–350 mg/dL with average plasma alanine aminotransferase (ALT) levels of 160 and 192 IU/L after one and two weeks, respectively, and reached ALT levels as high as 260–270 IU/L after three to four weeks of alcohol infusion [56].

Binge EtOH by oral gavage is another method of EtOH administration to animals. A single EtOH binge increases BAC in a dose- and time-dependent manner, with peak blood alcohol levels at 1–2 h post ingestion [24,57]. A range of EtOH doses (3–7 g/kg) was shown to cause a gradual increase in BAC levels in mice [24]. The highest BAC levels (~350–400 mg/dL) were observed from 1 h to 3 h after an EtOH binge (5 g/kg), which decreased substantially (<50 mg/dL) by 6 h and reached baseline by 9 h [58]. Further, mice exposed to a single EtOH binge (5 g/kg) daily for 5 or 10 consecutive days demonstrated BAC levels of 374 ± 22 mg/dL and 463 ± 25 mg/dL, respectively [59]. 

Administration of an EtOH binge further increases BAC levels in chronic EtOH-fed animals. In the NIAAA mouse model, *ad libitum* EtOH feeding for 10 days (5% *v*/*v*, 10 d) resulted in a BAC of ~180 mg/dL, but 10 days combined with a single binge (10 d + 1 B) induced BAC levels as high as ~400 mg/dL 1 h and 2 h following the binge bolus [20]. In mice fed an EtOH-containing liquid diet (5% EtOH) for two weeks and administered EtOH binges (5 g/kg) during the last three days, BAC was significantly increased 1 h post-binge in chronic-binge EtOH group compared to chronic EtOH alone (406 ± 76 mg/dL vs. 93 ± 69 mg/dL after one binge and 428 ± 84 mg/dL vs. 82 ± 43 mg/dL after three binges) [60]. Similarly, in a rat model [61], chronic EtOH feeding for four weeks (5% *v*/*v*) followed by either single (5 g/kg) or repeated binge EtOH administration (5 g/kg, three doses, 12 h intervals) significantly increased BAC levels (~40 mM (175.7 mg/dL) and ~120 mM (540.3 mg/dL) respectively) compared to chronic alcohol feeding alone (~20 mM (101.5 mg/dL)), and these increases were associated with augmented liver injury. In contrast, there are also studies that show unaltered BAC levels following chronic-binge EtOH feeding [58]. For example, Matyas et al. examined the BAC in mice 9 h after a single EtOH binge (5 g/kg) following *ad libitum* feeding of LD diet (5% *v*/*v*) for 10 days (10 d + 1 B) or 40 days (40 d + 4 binges) [58]. While the BAC was significantly increased in chronic and chronic-binge EtOH-fed groups compared to isocaloric diet-fed controls, single or multiple EtOH binge administration did not further increase BAC levels over those observed in chronic EtOH-fed groups. Blood EtOH levels were ~150–180 mg/dL in both 10 d and 10 d + 1 B groups, and ~50 mg/dL in 40 d and 40 d + 4 B groups. The reasons for the inconsistent effects of binge EtOH on BAC levels are not well understood but they are likely influenced by various factors such as time, dose, and route of EtOH gavage, time of post-binge euthanasia [20], animal handling, differences in housing environment and microbiota [62], or perhaps other unidentified factors. 

In summary, binge EtOH administration produces markedly elevated BAC levels, which is critical for achieving advanced liver injury. High BAC levels allow greater quantities of EtOH into the portal circulation, thereby exposing livers to higher EtOH concentrations. The liver also sustains the greatest degree of alcohol-induced tissue injury because it is the primary site of EtOH metabolism. Therefore, high BAC, coupled with the predominant metabolism by the liver, causes biochemical changes in liver cells that, at least in part, mediate and/or exacerbate ALD.

## 3. Rodents Models of ALD Utilizing Binge Ethanol Administration: Liver Injury Outcomes and Mechanistic Insights

**Figure 2:**
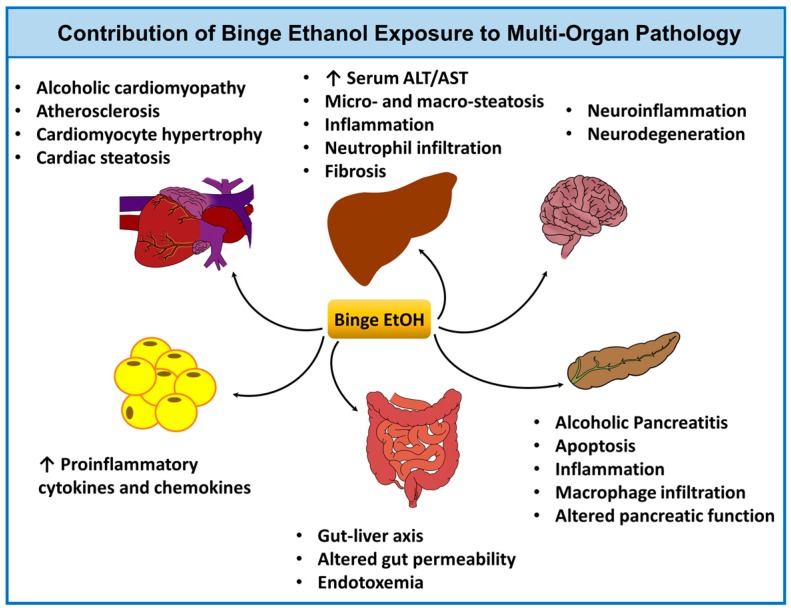
Binge ethanol and multi-organ pathology. Acute (binge) ethanol intoxication contributes to tissue injury in several organ systems including the liver, gut [63,64,65,66], heart [58,67,68,69,70,71], adipose [72], pancreas [73,74], and brain [75,76,77]. The potential pathophysiological consequences of binge drinking and its impact on each organ system are shown. Abbreviations: EtOH, ethanol; ALT, alanine aminotransferase; and AST, aspartate aminotransferase.

Historically, animal models of ALD utilizing binge EtOH administration have progressed from single or multiple binges alone (where animals are usually maintained on a standard rodent chow) to single or multiple binges combined with chronic EtOH exposure (where animals are provided *ad libitum* liquid alcohol containing diet). Several variations have been introduced to these models by multiple investigators, including different doses of EtOH for binge, dietary manipulations, or utilizing additional ‘second-hits’ (e.g., lipopolysaccharide (LPS)) to achieve greater liver injury and mimic distinct features of ALD as observed in humans. These models have also been used to examine EtOH-induced pathology of other organs, such as the heart, and pancreas (Figure 2), as well as to explore the interaction of the liver with the gut, adipose tissue, and the brain. In this section, we review selected studies utilizing EtOH binge(s) and provide liver injury outcomes and mechanistic insights as summarized in Table 1. 

### 3.1. Single or Multiple Ethanol Binge Administration to Rodents Maintained on Standard Chow Diet

Single or multiple EtOH binge administration causes acute alcohol-induced liver injury. For example, a single EtOH bolus (6 g/kg) in mice markedly increased serum ALT levels at 4 h post-binge, promoted hepatic microvesicular steatosis and inflammation, and caused significant injury to the ileum compared with isocaloric maltose-gavaged controls [25]. Epigenetic modification of critical proteins is implicated as one of the mechanisms underlying binge EtOH-mediated liver injury [78,79]. In a study from our laboratory, three EtOH binges (4.5 g/kg) at 12 h-intervals resulted in markedly increased plasma ALT activity, hepatocyte apoptosis, and microvesicular liver steatosis at 4 h post-final binge in male C57BL/6J mice [26]. This study demonstrated HDACs were important in the regulation of genes involved in hepatic fat metabolism. In a similar model (three EtOH binges, 12 h apart), a higher dose of EtOH bolus (6 g/kg) resulted in comparable effects on the liver in female mice [27]. Mechanistically, this study showed that liver damage resulting from multiple EtOH binges was associated with altered fat metabolism, increased hepatic oxidative stress and inflammation, intestinal injury and endotoxemia [27]. Given the importance of the gut–liver axis and the gut microbiota in chronic and acute ALD, a recent study from Dr. Schnabl’s laboratory subjected germ-free and conventional C57BL/6 mice (maintained on standard rodent chow) to a single EtOH binge (3 g/kg) [62]. Despite the fact that EtOH binge was associated with increased hepatic and intestinal expression of EtOH-metabolizing enzymes in germ-free mice leading to faster EtOH clearance from the blood and lower BAC, these mice had significantly greater liver injury, steatosis, and inflammation compared to conventional mice. This suggests that gut flora plays a protective role against liver injury. 

In addition, binge EtOH models have elucidated mechanistic differences underlying liver damage due to acute bolus of different alcoholic beverages, (e.g., plain EtOH vs beer) [80,81]. In these studies, female C57BL/6J mice received a single dose of EtOH solution (6 g/kg), isocaloric beer (EtOH content: 6 g/kg) or isocaloric maltodextrin solution [80,81]. Animals were euthanized 2 h or 12 h post-binge treatment [81]. Compared with plain EtOH, administration of a beer binge was associated with significantly less liver injury evidenced by plasma ALT, liver steatosis, Kupffer cell activation, as well as improved markers of intestinal barrier function. 

### 3.2. Rodents Models of Chronic Ethanol Feeding Combined with Binge EtOH Administration

Over the last decade, several murine models have been developed that combine chronic EtOH feeding (short- or long-term) with binge EtOH administration (single or multiple). Chronic-binge models cause more robust liver injury compared to *ad libitum* chronic feeding or EtOH binge alone and have been utilized for studying the mechanisms and risk factors (e.g., age, sex, and circadian disruption) underlying EtOH-induced multi-organ toxicity. 

#### 3.2.1. Short-Term Chronic Ethanol Feeding Combined with a Single EtOH Binge (Chronic-Plus-Binge NIAAA Model)

The original chronic-plus-binge model (known as “10 plus one” [10 d + 1 B] or the NIAAA model) was developed by Dr. Bin Gao’s group [20]. In this model, male and female C57BL/6 mice are fed with the Lieber–DeCarli EtOH diet *ad libitum* or pair-fed with an isocaloric control diet for 10 days (10 d, 5% EtOH *v*/*v*) and are gavaged with a single bolus of EtOH (5 g/kg, 20% EtOH) or isocaloric maltose dextrin in the early morning of day 11. This model demonstrated liver steatosis and injury, increased hepatic oxidative stress and pro-inflammatory cytokine production [20,28]. Chronic plus binge EtOH resulted in greater levels of serum transaminases (ALT and AST) compared to chronic (10 d) or single EtOH gavage [28]. Although serum transaminases were increased at both 6 h and 9 h post-binge, the peak increase was observed at 9 h post-binge, reaching approximately 250 IU/L (ALT) and 420 IU/L (AST) [28]. Notably, EtOH binge alone elevated hepatic triglyceride levels similar to the chronic-binge group [28]. Mechanistically, a single EtOH binge has been shown to induce neutrophil-mediated liver injury in chronic EtOH-fed mice via several processes including increased expression of E-selectin, natural killer T cell activation, and endoplasmic reticulum (ER) stress-dependent mitochondrial DNA-enriched microparticle release [20,82,83,84,85]. 

#### 3.2.2. Long-Term Chronic Ethanol Feeding Combined with Single or Multiple EtOH Binges

Longer-term variations of the 10 d + 1 B NIAAA model combined with single or multiple binges have recently been reported. For example, Xu et al. showed that combining single or multiple EtOH binges with long-term chronic EtOH feeding in mice recapitulated certain histological and molecular features of advanced clinical alcoholic steatohepatitis (ASH) [32]. In this study, male C57BL/6N mice were subjected to chronic EtOH feeding for 4, 8, or 12 weeks-plus-1 binge (4 w + 1 B, 8 w + 1 B, and 12 w + 1 B) or 8 weeks-plus-biweekly binges (8 w + nB). 8 w + 1 B provoked more severe elevation of serum transaminases, hepatic macrosteatosis, neutrophil infiltration, inflammation, and fibrosis when compared to 10 d + 1 B [32]. Mechanistically, FSP27/CIDEC was identified as an important mediator underlying ASH development [32]. Of note, multiple binges in the setting of chronic EtOH feeding resulted in significantly lower [20] or similar [32] serum transaminase levels compared to a single binge (10 d + 1 B). The authors suggested that a single EtOH binge represents an acute liver injury, which in general causes higher serum transaminase activity compared to chronic liver damage (note: multiple binges are considered to be chronic). 

Chronic binge EtOH models have also been developed in rats. For example, multiple EtOH binges (three doses, 5 g/kg, 12 h apart) were administered to rats after 4 weeks of chronic EtOH feeding (5% EtOH *w*/*v*). This paradigm causes greater plasma ALT levels, hepatic macro-vesicular steatosis, inflammation, and neutrophil infiltration compared to chronic EtOH feeding alone or multiple EtOH binges [61,86]. Further, this model has been widely utilized to elucidate epigenetic mechanisms contributing to liver injury in acute-on-chronic EtOH consumption settings [30,31]. 

#### 3.2.3. Application of Ethanol Binge Models to Study Multi-Organ Pathology and the Effects of Aging, Sex, and Circadian Rhythm

The liver is the main organ that metabolizes EtOH, and is therefore the primary target for alcohol-induced toxicity. However, other organs and systems, including the heart, pancreas, brain, lungs, gut, and the immune system are also adversely affected by chronic and acute alcohol consumption. It has been demonstrated that binge drinking is associated with adverse cardiovascular effects, including macro- and microvascular dysfunction [67], increased atherosclerotic plaque development [68], coronary calcification [69], and myocardial injury [58,70,71]. In addition, binge drinking leads to acute and recurrent pancreatitis [73,74], and neuropathology [75,76,77]. The chronic-plus-binge mouse models have found broad applications in examining the mechanisms of EtOH-induced multi-organ damage. For example, Matyas et al. employed variations of the NIAAA model (such as 10 d + 1 B, 20 d + 2 B, and 40 d + 4 B), and demonstrated that chronic-binge EtOH feeding in mice leads to alcoholic cardiomyopathies characterized by increased myocardial oxidative/nitrative stress, impaired mitochondrial function and biogenesis, cardiomyocyte hypertrophy, and enhanced cardiac steatosis [58]. Paradigms of binge EtOH exposure in rodents were utilized to study the impact of drinking patterns on alcoholic pancreatitis [59,60]. In these studies, male C57BL/6 mice were maintained on a standard rodent chow diet and administered EtOH binges (5 g/kg) daily for 10 days (10 B) [59], or were fed an EtOH liquid diet (5% EtOH) for two weeks with EtOH binges daily during the last three days (2 w + 3 B) [60]. The addition of EtOH binges caused a more severe (compared to standard chow or chronic EtOH feeding) spectrum of pancreatic injury as shown by significant apoptotic cell death, macrophage infiltration and increased inflammation, and altered pancreatic function via mechanisms involving increased oxidative and ER stress [60]. EtOH binges for 10 consecutive days (10 B; 5–6 g/kg) were also shown to cause neuroinflammation, activation of microglia and astrocytes, and neurodegeneration in mice in an age-specific (adolescent vs. adult) manner [87,88]. 

Aging is associated with increased risk of alcohol-induced liver injury in humans and rodent models [29,89,90,91,92,93]. In a study by Ramirez et al., young (8–12 weeks), middle-aged (12–14 months), and old (>16 months) female mice were subjected to 10d + 1B or 8 weeks + multiple binges (8w + nB) of EtOH administration [29]. Middle-aged and old mice were more susceptible to liver injury induced by both protocols as shown by greater induction of serum transaminases, higher degree of steatosis, increased hepatocyte apoptosis, hepatic neutrophil infiltration, and fibrosis compared to young mice [29]. Increased susceptibility to age-dependent liver injury was mediated by *SIRT1* down-regulation in both hepatocytes and hepatic stellate cells.

Similar to aging, females are at a greater risk of ALD compared to males [1,94,95,96]. Women (especially long-term drinkers) develop ALD after consuming less alcohol compared to men [1,64,94]. Similarly, sex differences were observed in rodent models of ALD [72,97,98,99]. The 10 d + 1 B protocol induced higher serum transaminases in female mice compared to males [28,72]. Fulham et al. demonstrated sex-dependent differences in hepatic triglycerides, pro-inflammatory cytokine, and chemokine expression in the liver and adipose tissue [72]. On the other hand, some studies from the Gao and Schnabl laboratories showed that average serum transaminase levels were comparable between male and female mice in the 10d + 1B model [20,100]. These discrepancies might be attributed to inter-experimental variations and suggest the need for careful consideration while interpreting experimental results. Further studies on sex differences in murine models are warranted.

Circadian clock disruption is an important contributor to alcohol-induced pathology [101,102,103,104,105,106,107,108]. Recent studies have established a link between alcohol consumption and impaired circadian rhythm in the liver [101,102,103,109,110,111,112]. The effect of binge EtOH on the liver circadian clock was examined by using the NIAAA 10d + 1B model in male wild-type (WT) C57BL/6 and small heterodimer partner (SHP)-deficient mice [111,112]. SHP is a mediator of multiple circadian metabolic effects [112]. Metabolomics analysis revealed that EtOH binge and SHP deficiency caused distinct circadian changes in metabolites from pathways of carbohydrates, lipids, pentose phosphate, amino acids, nucleotides, and tricarboxylic acid cycle in the liver and serum [111]. The expression of lipid synthesis genes and ER stress markers also exhibited distinct variations in response to EtOH binge [108]. Further, a crucial crosstalk was shown to exist between SHP and Rev-Erbα, two important liver clock modulators, and that crosstalk mediates alcoholic steatosis via the transcription factor C/EBP-homologous protein (CHOP) [108].

#### 3.2.4. Chronic Intragastric Ethanol Feeding Combined with Multiple Ethanol Binges (Tsukamoto–French Hybrid Model)

As highlighted previously, greater liver injury has been achieved by modifying the route of EtOH delivery from *ad libitum* (Lieber–DeCarli model) to direct continuous IG EtOH infusion (the Tsukamoto–French model). Dr. Tsukamoto’s group has recently modified the model by incorporating EtOH binges into the paradigm. They developed a hybrid model where animals were fed a Western diet (high in cholesterol and saturated fat) for two weeks followed by IG infusion of EtOH (up to 27 g/kg/day) with a high fat liquid diet (corn oil-enriched) for eight weeks and weekly binges of EtOH (~4–5 g/kg) from the second week of IG infusion [33]. This protocol induced severe liver damage, wherein repeated EtOH binge administration mediated a transition from chronic ASH (macrophage-mediated liver injury and inflammation with perisinusoidal and pericelluar fibrosis) to frank AH (neutrophil-mediated liver damage), with clinical features such as hypoalbuminemia, bilirubinemia, and splenomegaly.

### 3.3. Rodent Models of High Fat Diet-Induced Liver Injury Combined with Binge Ethanol Administration

The initiation and progression of ALD is strongly affected by the presence of comorbid conditions (e.g., obesity). Obese alcoholic subjects have increased serum ALT levels and higher risk of developing steatohepatitis, cirrhosis, and hepatocellular carcinoma as compared to non-obese alcoholics or obese non-alcoholics [113,114,115,116,117,118,119,120]. This section outlines selected studies that have addressed the interaction of binge EtOH and obesity using rodent models of diet-induced or genetic obesity. 

Chang et al. described a simple two-hit model to show that short- or long-term HFD feeding plus a single EtOH binge synergistically induces marked elevation of serum ALT and produces features of severe steatohepatitis in mice [36]. In this study, C57BL/6 mice were fed HFD (60% kcal fat) for three days or three months followed by a single gavage with EtOH (5 g/kg) or isocaloric maltodextrin solution. Serum transaminase levels and hepatic neutrophil infiltration were markedly increased in three-day- and three-month-HFD + binge EtOH groups (3d-HFD + 1B; 3 m-HFD + 1B), with greater induction in the long-term group. Neutrophil infiltration was observed as early as 3 h-post binge in the 3 m-HFD + 1 B cohort, with highest levels occurring at 6 h and 9 h-post binge, and substantial neutrophil infiltration was still observed 24 h-post binge which declined by 48h. Mechanistically, acute EtOH binge-mediated upregulation of the hepatic pro-inflammatory cytokine, CXCL1 promoted neutrophil infiltration in mice subjected to either short or long term HFD feeding. This is a valuable model that mimics acute ASH in obese binge drinkers. Nieto et al. used a unique diet-alcohol combination to study the effects of whiskey binge drinking on fatty liver in rats fed a choline-deficient (CD) diet [35]. Rats were gavaged with 1.5 mL/100 g of commercial whiskey three times per week for three months and were euthanized 72 h post-final binge. Whiskey binges in rats fed the CD diet resulted in induction of serum transaminases, hepatic microsteatosis, and triggered liver apoptosis and fibrosis over that found in rats fed the CD diet alone.

Binge EtOH administration is also detrimental in animal models of genetic obesity. For instance, Carmiel-Haggai et al. showed that an EtOH binge of 4 g/kg every 12 h for three days induced serum ALT activity, hepatic steatosis, and inflammation in genetically obese *fa*/*fa* Zucker rats compared to EtOH-binged lean littermates [34]. Liver injury was mediated through a mechanism involving oxidative and nitrosative damage. Collectively, these observations show that binge alcohol intake is an important participant in the progression from fatty liver to advanced ASH in obese states, suggesting that obese binge drinkers may be at an increased risk of developing more severe liver injury.

### 3.4. Rodent Model of Combined Binge Ethanol and Lipopolysaccharide Administration

Both chronic and acute EtOH consumption causes endotoxemia (elevated blood levels of bacteria-derived products, e.g., LPS) in humans and experimental animals [63,64,65,66,121]. A single alcohol binge in healthy subjects was shown to cause a rapid and transient increase in serum LPS levels [63]. LPS plays an important role in initiating and promoting ALD by inducing inflammation through toll-like receptor 4 expressed on both parenchymal and non-parenchymal cells in the liver [122]. Beier et al. developed a paradigm combining multiple EtOH binges and LPS administration as a model to elucidate the effects of EtOH pretreatment on LPS-mediated liver injury [37]. Male C57BL/6J mice were subjected to an EtOH gavage (6 g/kg) or isocaloric/isovolumetric maltodextrin solution for three consecutive days after which LPS (10 mg/kg, i.p.) was injected 24 h following the last EtOH bolus. Animals were euthanized at various time-points from 6 h to 48 h after LPS injection. This model revealed that EtOH-LPS treatment causes more severe liver injury compared to EtOH or LPS alone with markedly increased plasma ALT and AST levels (~400 U/L), necro-inflammatory foci and hepatic neutrophil infiltration at 24 h-post LPS administration. A marked increase in hepatic fibrin deposition mediated by inhibition of fibrinolysis by PAI-1 was observed in EtOH-LPS-treated mice. This is a short-term model which is relatively easy to perform and which allows for the examination of molecular mechanisms underlying liver pathology caused by acute EtOH and LPS exposure. 

### 3.5. Technical Considerations

There are several critical factors that may influence the effects of EtOH binge administration, including, for example, the dose of EtOH, the number of binges, the concentration of EtOH in the solution used for the gavage, and the time point(s) of sample collection. The control solution for EtOH bolus is also an important consideration. Maltodextrin remains the more widely-used control rather than water or saline. It enables the maintenance of isocaloric intake in all animals (controls and EtOH-binged) by substituting the calories gained from EtOH intake by those provided by maltodextrin. However, maltodextrin administration might cause distinct metabolic abnormalities [123], which currently receive little attention. The strain of mice is also an important consideration. There have been no systematic studies that have addressed strain differences in binge EtOH-mediated liver injury in rodents. However, marked strain differences have been demonstrated in alcohol-induced liver damage and mechanistic pathways in mice using other EtOH administration paradigms [124,125]. These factors should be considered when designing and interpreting rodent models of EtOH binge paradigms.

## 4. Summary and Conclusions

Alcohol has been administered to rodents by a variety of different paradigms, each having their respective strengths, limitations, and applications. Incorporating binge EtOH administration into traditional animal models of ALD (e.g., *ad libitum* chronic EtOH feeding) causes more severe liver pathology. It closely resembles the drinking behavior in humans, and produces many of the histological and molecular features of alcohol-induced liver injury seen in patients with ALD. Several binge EtOH animal models have been recently developed, including administration of single or repeated EtOH binges alone or in combination with chronic EtOH exposure, HFD feeding, or LPS challenge. In particular, the recent NIH NIAAA paradigm (single or multiple EtOH binges combined with chronic EtOH) has been extensively utilized by numerous investigators to examine alcohol-induced liver and multi-organ pathology. 

However, there is no perfect model that recapitulates all aspects of human ALD. Single binge models represent acute alcoholic liver injury and induces only mild steatosis, but is quick and relatively easy-to-perform. Chronic-plus-binge EtOH models represent a mixture of acute-on-chronic and chronic liver injury often seen in patients with AH and produces steatohepatitis (steatosis and inflammation) and mild fibrosis. However, this model can be long-term and laborious. The recapitulation of alcohol-induced fibrosis in rodent models is more challenging than steatosis and inflammation. The hybrid model of HFD and IG EtOH feeding with weekly EtOH binges produces liver fibrosis, further supporting the utility of binge EtOH paradigms. Binge EtOH-plus-HFD model allows to examine the effect of comorbidities such as obesity on the progression of ALD. Therefore, the careful choice of experimental model is vital, and should be based on the specific scientific inquiry (acute or chronic EtOH exposure), the human liver injury feature to be simulated (steatosis, inflammation, and/or fibrosis), and the technical expertise available. Taken together, binge EtOH models are attractive tools for identifying possible novel mechanisms, diagnostic or prognostic biomarkers, and therapeutic targets for ALD.

## Figures and Tables

**Table 1:** Experimental studies showing the effect of binge EtOH administration paradigms on liver injury outcomes in rodent models *.

Strain	Age	Sex	Binge Model	Liver Injury Outcomes and Mechanistic Insights	Comments	Ref.
**EtOH binge alone (single or *n*; *ad libitum* standard chow diet)**						
C57BL/6 x C3HF1 mice	8 weeks	Females	1 binge (3, 4, 5, 6, and 7 g/kg)	↑ ALT with 7 g/kg EtOH dose (4 and 12 h post-binge)	Shows dose- and time-dependent relationship of binge with BAC and ALT	[24]
129/Sv^PC^ J mice	8–10 weeks	Males	1 binge (6 g/kg)	↑ ALT, AST microsteatosis, mild necrosis ↑ liver TNF-α ↑ plasma endotoxin (1.5 h post-binge), injury to the ileum, and ↑ intestinal permeability	Shows effect of single binge on liver injury, and intestinal permeability	[25]
C57BL/6J mice	8 weeks	Males	3 binges (4.5 g/kg) at 12 h intervals	↑ ALT microsteatosis ↑ hepatocyte apoptosis ↓ hepatic *Hdac* (1, 7, 9, 10 and 11) ↑ hepatic *Hdac 3* ↓ Cpt1a and ↑ Fas expression	Shows effect of multiple binges on liver injury, and HDAC as a mechanism	[26]
Mice on 129/Svj background		Females	3 binges (6 g/kg) at 12 h intervals	↑ ALT (6 h-post binge) ↑ hepatic triglycerides ↑ hepatic pro-inflammatory cytokines ↑ hepatocyte apoptosis ↑ hepatic oxidative stress ↑ serum endotoxin ↑ injury to the ileum	Shows effect of multiple binges on liver injury, and intestinal permeability in female mice	[27]
**Chronic (short- or long-term) EtOH diet plus EtOH binge (single or multiple)**						
C57BL/6N mice	8–10 weeks	Males	10 d + 1 B (5 g/kg)	↑ ALT, AST (6 h and 9 h; females > males) ↑ liver and serum triglycerides ↑ hepatic oxidative stress ↑ hepatic lipogenesis ↓ hepatic fat oxidation	Shows effect of NIAAA 10 d + 1 B model on liver injury in male mice	[28]
C57BL/6J mice	8–12 weeks	Females	10 d + 1 B (5 g/kg)	↑ ALT, AST ↑ hepatic inflammation ↑ hepatic neutrophil infiltration and activation ↑ hepatic E-selectin, SELP, ICAM-1, ↓ VCAM-1	Shows effect of 10 d + 1 B model on liver injury in female mice, and hepatic neutrophils as a mechanism	[20]
C57BL/6N mice	Young (y; 8–12 weeks), Middle-age (m; 12–14 months), Old (o; > 16 months)	Females	10 d + 1 B (5 g/kg) 8 w + *n*B	**10 d + 1 B**: y < m < o mice ↑ ALT, AST ↑ hepatic triglycerides ↑ hepatocyte apoptosis ↑ hepatic neutrophil infiltration ↑ hepatic fibrosis ↓ hepatic SIRT1 expression **8 w + *n*B**: y < m < o mice ↑ ALT, AST ↑ hepatic triglycerides ↑ hepatocyte apoptosis, ↓ liver regeneration ↑ hepatic oxidative stress ↑ hepatic fibrosis, ↑ α-SMA protein, and ↑ mRNAs for *α-Sma*, *collagen I* and *III* in aged mice	Shows effect of long-term chronic EtOH + multiple binges in mice; Examines effect of aging; Shows that model also recapitulates features of AH and achieves hepatic fibrosis	[29]
C57BL/6J mice	7 weeks	Males	Chronic 4 weeks + 3 binges (3.5 g/kg) at 12 h intervals	↑ ALT macrosteatosis ↑ hepatic triglycerides ↑ CYP2E1 protein ↑ hepatic adenosine ↑ hepatic histone modification (phosphorylation, dimethylation and acetylation) ↑ hepatic GCN5 protein ↑ hepatic HDAC3 protein	Shows effect of long-term chronic EtOH + multiple binges in mice; Shows histone modification as a common mechanism similar to single binge alone	[30]
Sprague Dawley rats	7 weeks	Males	Chronic 4 weeks + 3 binges (5 g/kg) at 12 h intervals	↑ hepatic histone modification (phosphorylation, acetylation and dimethylation) ↑ hepatic GCN5 protein ↑ hepatic HDAC3 protein ↑ hepatic ERK phosphorylation	Shows effect of long-term chronic EtOH + multiple binges in rats; Shows histone modification as a common mechanism	[31]
C57BL/6N mice	8–10 weeks	Males and Females	1 binge 10 d + 1 B Chronic 4, 8, and 12 weeks + 1 binge (E8 w; E4 w + 1 B; E8 w + 1 B; E12 w + 1 B) Chronic 8 weeks + biweekly binges (5 g/kg; E8 w + nB)	↑ ALT (E12 w + 1 B, E8 w + 1 B > E4 w + 1 B > E10 d + 1 B, E8 w + nB) ↑ hepatic macro- and microsteatosis (E8 w + 1 B, E12 w + 1 B, E8 w + nB > E8 w) **E8 w + 1 B, E8 w + nB > E8 w** ↑ hepatic steatosis, apoptosis, oxidative stress, and inflammation ↑ hepatic neutrophil infiltration ↑ fibrosis (αSMA, Sirius red, collagen expression), ↑ keratin 8 and keratin 18 expression (~4- to 8-fold) ↑ *Fsp27* gene and protein ↑ PPARγ and active nuclear form of CREBH-N protein, ↑ CHOP and BiP (**E8 w + 1 B**)	Shows effect of varying lengths of long-term chronic EtOH + single/multiple binges; Shows model achieves fibrosis and recapitulates advanced ALD	[32]
C57BL/6 mice		Males	Hybrid model: *ad libitum* Western diet (cholesterol and saturated fat; HCFD, 2 weeks) combined with IG EtOH liquid diet (27 g/kg/day, 8 weeks) + weekly EtOH binges (4~5 g/kg) started from second week of IG feeding	severe ASH with MNC inflammation, PMN infiltration hepatomegaly ↑ ALT, AST ↑ hepatic steatosis splenomegaly, hypoalbuminemia, and hyperbilirubinemia ↑ plasma endotoxin ↑ liver fibrosis	Shows model recapitulates features of advanced ASH	[33]
**High-fat diet plus EtOH binge (single or *multiple*)**						
fa/fa obese Zucker rats	15 weeks	Males	Binges (4 g/kg) every 12 h for 3 days	↑ ALT micro and macrovesicular steatosis hepatocyte ballooning and inflammation impaired antioxidant capacity ↑ lipid peroxidation ↑ hepatic apoptosis	Shows effect of multiple binges in a model of genetic obesity in rats	[34]
Lewis rats	12 weeks	Males	Choline-deficient diet + 3 binges of whiskey (1.5 mL/100 g) per week for 3 months	↑ transaminases ↑ microvesicular steatosis and apoptosis liver fibrosis: ↑ collagen, α-Sma, Hsp47 protein	Shows effect of multiple whiskey binges in a model of diet-induced obesity in rats	[35]
C57BL/6J mice	8–12 weeks	Males	HFD (60% kcal fat) for 3 days or 3 months + 1 binge (5 g/kg) [3 d-HFD + 1 B; 3 m-HFD + 1 B]	**3 m-HFD + 1 B > 3 d-HFD + 1 B > 3 d/3 m-HFD alone** ↑ ALT/AST ↑ hepatic neutrophil infiltration ↑ hepatic inflammation *Cxcl1* expression in hepatocytes, hepatic stellate cells and sinusoidal endothelial cells, and adipose tissue ↑ serum CXCL1	Shows effect of a single binge on short- or long-term HFD-induced obesity	[36]
**Multiple EtOH binges plus lipopolysaccharide injection**						
C57BL/6J mice	8 weeks	Males	3 binges (6 g/kg) in 3 days + LPS (10 mg/kg i.p.) 24 h post-final binge	↑ ALT and AST (24 h-post LPS) ↑ hepatic triglycerides (12 h-post LPS) ↑ hepatic neutrophil infiltration (24 h-post LPS) ↑ hepatic fibrin deposition (24 h-post LPS) ↑ hepatic PAI-1 expression (4 h- and 24-post LPS) hepatic TNFα expression (No effect at 4 h and ↓ at 24 h-post LPS)	Shows effect of multiple binges combined with LPS injection	[37]

* The selected studies include different rodent models of binge EtOH administration-induced alcoholic liver disease (e.g., binge alone, or in combination with *ad libitum* EtOH feeding, HFD, or LPS injection). Abbreviations: ALT: alanine aminotransferase; AST: aspartate aminotransferase; ASH: alcoholic steatohepatitis; α-SMA: α smooth muscle Actin; BAC: blood alcohol concentration; BiP: binding immunoglobulin protein; Cpt1: carnitine palmitoyltransferase I; CHOP: C/EBP-homologous protein; CREBH-N: cyclic AMP response element-binding protein H; ERK: extracellular signal-regulated kinase; EtOH: ethanol; Fas: fatty acid synthase; Fsp27: fat-specific protein 27; GCN5: histone acetyltransferase; Hdac: histone deacetylases; Hsp47: heat shock protein 47; ICAM-1: intercellular Adhesion Molecule 1; IG: intragastric; LPS: lipopolysaccharide; MNC: mononuclear cell; PAI-1: plasminogen activator inhibitor-1; PMN: polymorphonuclear cell; PPAR-γ: peroxisome proliferator-activated receptor γ; SIRT1: sirtuin 1; TNFα: tumor necrosis factor α; SELP: P-selectin; VCAM-1: vascular cell adhesion molecule 1; 10 d + 1 B: 10 days chronic EtOH + 1 binge; 8 w + nB: 8 weeks chronic EtOH + multiple binges.
